# Supplementary material for: Evaluating the Physiologic Effects of Alfaxalone, Dexmedetomidine, and Midazolam Combinations in Common Blue-Tongued Skinks (Tiliqua scincoides)
Source: Animals (Basel). 2024 Sep 11;14(18):2636. doi: 10.3390/ani14182636 (PMC11429061; doi:10.3390/ani14182636)
Supplement: Supplementary file 1 [file animals-14-02636-s001.zip › animals-3165383-supplementary.pdf]

Table S1. Descriptive statistics for heart rate, respiratory rate, and blood gas parameters (pH, PCO_2, HCO_3, and lactate) measured in 11 common blue-tongued skinks provided four different combinations of sedatives: alfaxalone (A), alfaxalone-midazolam (AM), dexmedetomidine-midazolam (DM), and alfaxalone-dexmedetomidine-midazolam (ADM). Heart rates and respiratory rates are presented as median (interquartile range), and blood gas parameters as mean ± standard deviation.

**Variables**		**A**	**AM**	**DM**	**ADM**
Heart rate‡†*	Baseline	80 [72-84]	80 [76-84]	80 [72-84]	80 [80-88]
	Lowest	56 [52-56]	56 [48-64]	24 [20-32]	28 [26-32]
Respiratory rate‡†*	Baseline	36 [24-47]	27 [20-35]	29 [19-40]	23 [18-39]
	Lowest	9 [5-13]	7 [3-13]	0 [0-1]	0 [0-2]
pH†	Baseline	7.39 ± 0.12	7.39 ± 0.16	7.50 ± 0.04	7.40 ± 0.12
	Full sedation	7.31 ± 0.19	7.30 ± 0.11	7.27 ± 0.10	7.21 ± 0.12
	Recovery	7.42 ± 0.19	7.43 ± 0.07	7.39 ± 0.14	7.50 ± 0.06
PCO_2†*	Baseline	31.01 ± 10.89	32.25 ± 4.86	27.07 ± 1.07	27.17 ± 1.62
	Full sedation	32.09 ± 10.06	34.33 ± 3.65	37.70 ± 6.77	43.17 ± 6.88
	Recovery	28.96 ± 4.24	30.22 ± 2.72	31.80 ± 6.51	23.73 ± 3.70
HCO_3	Baseline	19.48 ± 3.38	21.32 ± 4.77	22.50 ± 2.38	18.84 ± 4.73
	Full sedation	17.58 ± 4.19	19.13 ± 5.37	18.75 ± 2.74	18.31 ± 3.35
	Recovery	20.90 ± 6.42	21.61 ± 3.94	20.63 ± 2.69	19.78 ± 2.15
Lactate†	Baseline	5.96 ± 4.07	4.17 ± 2.22	2.10 ± 1.57	4.41 ± 4.36
	Full sedation	9.07 ± 4.46	8.14 ± 3.60	8.15 ± 3.70	8.75 ± 3.67
	Recovery	5.54 ± 3.35	4.61 ± 2.49	7.21 ± 3.62	6.66 ± 1.05

The significant difference ($p<0.05$) confirmed by drug (‡), time (†), and drug×time (*).
